# Gold Quantum Rods Emit in the Shortwave Infrared (1000–2200 nm) with 10^4–5^ M^–1^ cm^–1^ Ultrabrightness

**DOI:** 10.1021/jacs.6c02971

**Published:** 2026-03-18

**Authors:** Lianshun Luo, Guiying He, Zhongyu Liu, Avirup Sardar, Weijie Ji, Abhrojyoti Mazumder, Sihan Chen, Qi Li, Rongchao Jin

**Affiliations:** † Department of Chemistry, 6612 Carnegie Mellon University, Pittsburgh, Pennsylvania 15213, United States

## Abstract

Fluorescence in the near-infrared-II (NIR-II) region (1000–2500 nm, also called shortwave infrared, SWIR) is highly attractive for advanced optical applications, including quantum photonics, biomedicine, and environmental sensing, yet the development of bright, stable, and tunable SWIR emitters remains a significant challenge. Here, we report ultrabright, subnanosecond fluorescence emission spanning 1000 to 2200 nm from a periodic series of atomically precise gold quantum rods (Au QRs) with quantum yields reaching up to ∼21% by suppressing singlet-to-triplet exciton conversion and electron–vibration coupling. The high quantum yields, together with high absorption coefficients (ε_peak_ ∼ 10^5–6^ M^–1^ cm^–1^) of Au QRs, offer unprecedented brightness (10^4–5^ M^–1^ cm^–1^), exceeding the state-of-the-art SWIR organic dyes by more than 2 orders of magnitude in the 1100–1400 nm region and 4 orders of magnitude in the >1500 nm region. Furthermore, Au QRs are successfully transferred into aqueous phase using a Pluronic F-127 amphiphilic polymer wrapping method. The results establish atomically precise Au QRs as a new benchmark platform that redefines the brightness and spectral reach attainable in SWIR fluorescence, providing a foundation for next-generation SWIR photonic and biorelated technologies.

## Graphical abstract

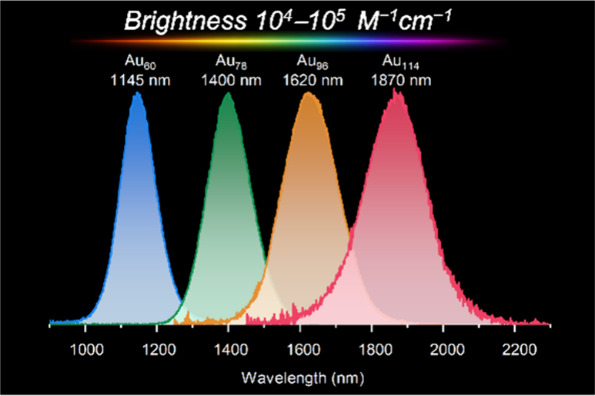

Fluorescence in the NIR-II or SWIR with wavelengths in the 1000–2500 nm range is important for many technological applications, including photonic quantum computing,^1^ quantum telecommunication,^2^ optogenetics,^3^ biomedical imaging,^4,5^ and cancer surgery and therapy.^6,7^ The key advantages of SWIR are reduced photon scattering by medium- and low-loss transmission, high atmospheric transparency, and minimal background noise. For example, SWIR is highly attractive in biomedical imaging, as SWIR light can penetrate much deeper than does visible light into biological tissues owing to much less light-scattering loss.^4,5^ This advantage, plus the much less autofluorescence background in the SWIR region from biological tissues, makes SWIR fluorescence particularly promising for imaging-guided cancer surgery and therapy.^8^

The design of bright SWIR emitters has only been met with limited success due to several fundamental challenges.^9–14^ First of all, fluorescence emission drastically diminishes in the SWIR region due to the energy gap law, which scales with 1/λ^3^ (where λ = wavelength).^9,15^ Second, high brightness (γ) requires both high quantum yield (QY) and large absorption coefficient (ε_λ_), i.e., γ = QY × ε_λ_,^14,16^ but the absorption coefficient diminishes rapidly at longer wavelengths due to enhanced thermal effects and electron–vibration coupling.^10,13^ To overcome these challenges, we aim to design low-dimensional gold quantum structures with anisotropic excitons for tailoring of absorptivity and electron–vibration coupling.^17^ In addition, for imaging and light-emitting applications,^13,18,19^ fast emission (i.e., fluorescence with nano- or picosecond (ns or ps) lifetimes) is highly desired. To suppress phosphorescence (typically microsecond lifetimes), one should develop strategies to inhibit intersystem crossing (ISC) from the singlet to triplet states, because the generation of phosphorescence requires an efficient ISC to populate the triplet state.^17^ According to the perturbation theory, the ISC rate is proportional to the spin–orbit coupling (i.e., heavy atoms tend to be strong in it) but inversely proportional to the singlet–triplet energy gap.^20,21^ By manipulating these two factors, we expect that the ISC process in low-dimensional Au quantum structures may be inhibited. Thus, with the suppressed ISC and electron–vibration coupling,^13^ we rationalize that it should be promising to construct bright SWIR (or NIR-II) emitters.

Herein we report a success in creating ultrabright, sub-ns, tunable SWIR fluorescence spanning 1000–2200 nm (edge) from a periodic series of gold QRs with high quantum yields (Φ up to 21%) and high brightness (γ ∼ 10^4–5^ M^–1^ cm^–1^), exceeding the state-of-the-art SWIR organic dyes by 2 orders of magnitude and meanwhile extending the emission range well beyond the ∼1500 nm telecom band with an ultrabrightness ∼ 10^4^ M^–1^ cm^–1^. By engineering the excited-state dynamics through structural control of the QRs, ISC and electron–vibration coupling are simultaneously suppressed, leading to sub-ns fluorescence. Furthermore, we have transferred Au QRs into an aqueous solution with their photoluminescence (PL) efficiency retained. The success in the design of an ultrabright SWIR emitter overcomes the key limitations of existing SWIR emitters in both brightness and spectral range, which will advance not only biomedical imaging/cancer therapy but also the photonic quantum technologies, light-emitting, and SWIR solar energy (1000–2500 nm) utilization via photon upconversion.

The series of Au QRs were synthesized in solution (see Supporting Information for details) and purified by thin-layer chromatography (Figure S1),^22^ including Au_60_(PET)_44_, Au_78_(PET)_56_, Au_96_(PET)_68_, and Au_114_(PET)_80_ (abbreviated Au_60_, Au_78_, Au_96_, and Au_114_), all protected by 2-phenylethanethiolate (PET) ligands. These Au QRs share a hexagonal close-packed kernel with a constant three-atom diameter (3.15 Å) and exhibit stepwise elongation to lengths up to ∼6 nm by periodic addition of Au_18_(PET)_12_ units (Figure 1A).^22^ Such structures, with a constant diameter and gradually increasing length, impart unique optical properties to the QRs, distinguishing them from other Au NCs^23^ in that the optical absorption spectra of Au QRs (Figure 1B) exhibit a constant peak at ∼400 nm and a much stronger, rod-length-dependent SWIR absorption band from 950 to 2100 nm (edge-to-edge). Notably, the molar absorption coefficients (ε_λ_) in the SWIR region is very high, ranging from 2.97 × 10^5^ to 9.06 × 10^5^ M^–1^ cm^–1^, which are 2 orders of magnitude higher than those of typical NCs (*ε* = 10^3–4^ M^–1^ cm^–1^).^23^ The strong SWIR absorption is attributed to the highly polarized HOMO–LUMO transition occurring predominantly along the longitudinal axis of the QRs.^22,24^ The intense and tunable SWIR absorption hints at the potential of these QRs as efficient, tunable SWIR emitters.

**Figure 1:**
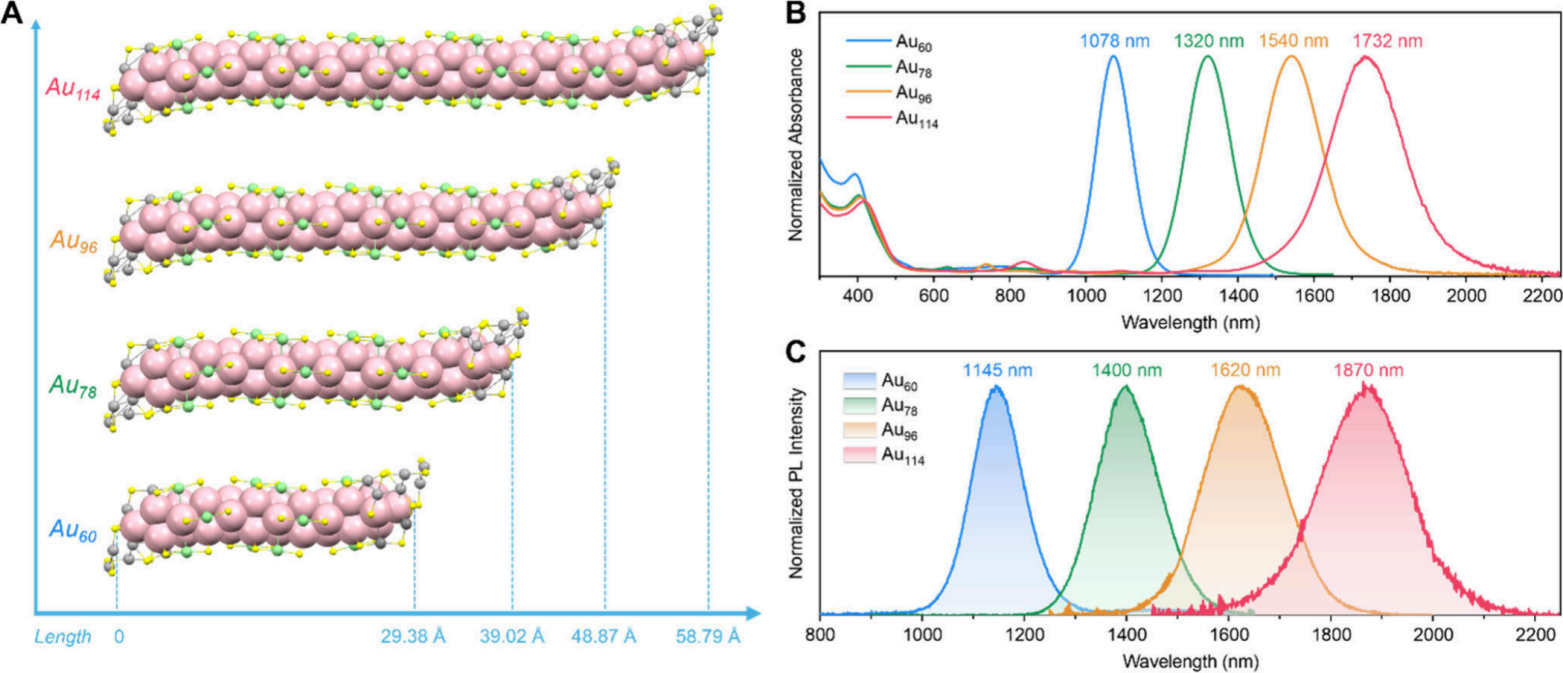
(A) Structures of Au_60_, Au_78_, Au_96_, and Au_114_ with kernel lengths ranging from 29.38 to 58.79 Å. Color code: yellow = S, other colors = Au, carbon tails are omitted for clarity. (B) Normalized optical absorption spectra of Au QRs (dissolved in tetrachloroethylene to avoid overtone vibrational peaks in the SWIR range). (C) Normalized PL spectra of Au QRs in toluene-*d*_8_. For excitation: 1078 nm for Au_60_, 1320 nm for Au_78_, 1540 nm for Au_96_, and 1700 nm for Au_114_ (note that the available excitation wavelength is limited to <1700 nm). The PL spectra of Au_60_ and Au_78_ were measured by a photomultiplier detector (PMT-1700, up to 1650 nm) and their QYs by an integrating sphere. The PL spectra of Au_96_ and Au_114_ were measured by an InAs photovoltaic detector (up to ∼3000 nm), and their QYs by a relative method (Figure S2) using Au_78_ as a reference.

It is worth noting that the optical absorption spectra of Au QRs resemble those of (bacterio)­chlorophylls. Specifically, the ∼400 nm peak is reminiscent of the Soret band in chlorophylls.^25^ This peak in Au QRs involves an isotropic excitonic transition, while the strong SWIR peak arises from an anisotropic exciton with the transition dipole moment mainly polarized along the rod’s long axis,^22^ analogous to the Q transition in chlorophyll.^25^ The anisotropic exciton is a consequence of the one-dimensional geometry of QRs and gives rise to tunable and intense SWIR absorption that scales with the rod length.

While some Au NCs have been reported to emit across the visible to NIR-I (700–1000 nm),^5,26–33^ it remains a challenge to extend into NIR-II or SWIR due to significantly stronger undesired nonradiative decay processes.^34^ The small optical gaps (*E*_g_) of Au QRs, e.g., 1.01 eV for Au_60_, 0.81 eV for Au_78_, 0.69 eV for Au_96_, and 0.60 eV for Au_114_, combined with their strong SWIR absorption, indicate their potential as efficient and tunable emitters in the technologically important SWIR range. The PL spectra of Au QRs are shown in Figure 1C. All QRs display SWIR emission, which progressively red-shifts with the rod length increase (or decreasing *E*_g_). The PL of the QRs indicates their small electron–vibration coupling (implied by the extremely narrow full-width at half maximum (fwhm) being ≤ 0.1 eV), and the Stokes shift (∼60 meV) is half reduced compared to that of the dual-emissive Au_42_ QR (∼120 meV),^17^ indicating more rigid structures of the longer QRs.

The QY of Au_60_ is 20.9% under 1078 nm excitation (Figure 1C and Figure S3), offering an extraordinary brightness of 6.2 × 10^4^ M^–1^ cm^–1^ (ε_1078_ = 2.97 × 10^5^ M^–1^ cm^–1^). The QY of Au_78_ is 15.8% at 1320 nm excitation (Figure 1C and Figure S4), with a brightness of 9.6 × 10^4^ M^–1^ cm^–1^ (ε_1320_ = 6.08 × 10^5^ M^–1^ cm^–1^). Similarly, Au_96_ and Au_114_ display QYs of 1.9% and 1.0%, yielding a brightness of 1.7 × 10^4^ M^–1^ cm^–1^ and 6.1 × 10^3^ M^–1^ cm^–1^, respectively (Au_96_ ε_1540_ = 9.06 × 10^5^ M^–1^ cm^–1^; Au_114_ ε_1700_ = 6.08 × 10^5^ M^–1^ cm^–1^, note that 1700 nm is the longest available excitation wavelength, which is slightly below the absorption peak wavelength of 1732 nm). Additionally, the PL excitation spectra of Au QRs (Figure S5) overlap well with their absorption spectra, indicating that the observed PL arises from their intrinsic electronic transitions. All of these QRs show excellent photostability, as evidenced by their unchanged absorption spectra before/after PL measurements (Figure S6). These QRs are also stable in solution or solid state (and redispersion).^22^

The PL lifetimes of Au QRs were measured to be 0.90 ns for the 1145 nm emission peak of Au_60_ (Figure 2A) and 0.80 ns for the 1400 nm emission peak of Au_78_ (Figure 2B), together with the insensitivity to O_2_ (Figure S7), revealing their fluorescent nature; note that the emission lifetimes of Au_96_ and Au_114_ could not be measured by the photomultiplier detector, but we expect the latter two to be subnanosecond lifetimes, too. The optical parameters of Au QRs are listed in Table 1.

**Figure 2:**
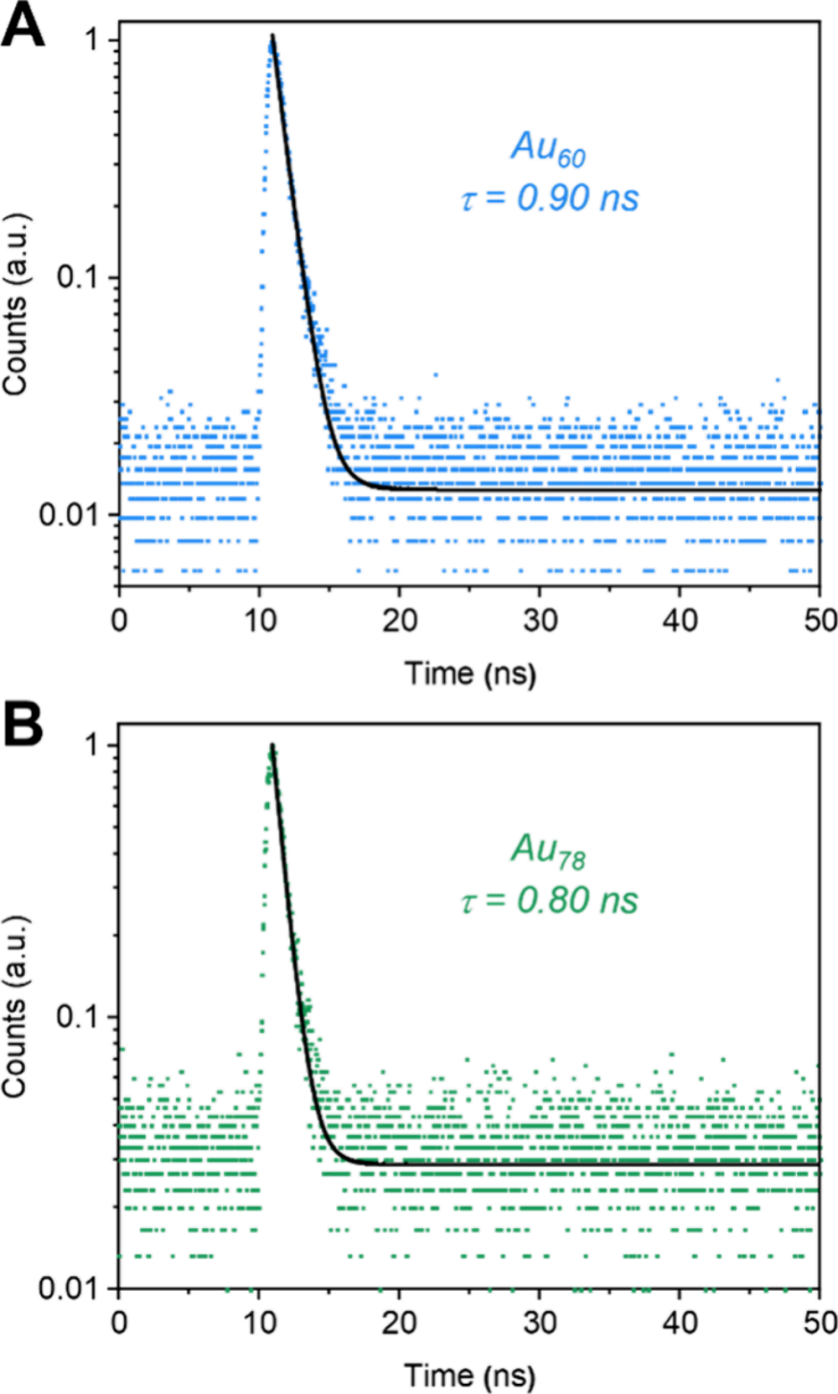
(A) Decay profile of the 1145 nm emission of the Au_60_ QR. (B) Decay profile of the 1400 nm emission of Au_78_ QR. Excitation: ∼80 ps pulsed laser (450 nm), instrument response function (IRF): ∼400 ps. The y-axis values are normalized counts.

**Table 1:** Absorption and Fluorescence Parameters of the Au QRs

Au QRs	λ_abs_ (nm)	ε (M^–1^ cm^–1^)	λ_em_ (nm)	QY (%)	Brightness (M^–1^ cm^–1^)	τ (ns)	fwhm_em_(nm, eV)
Au_60_(PET)_44_	1078	2.97 × 10^5^	1145	20.9	6.2 × 10^4^	0.9	(115, 0.108)
Au_78_(PET)_56_	1320	6.08 × 10^5^	1400	15.8	9.6 × 10^4^	0.8	(146, 0.092)
Au_96_(PET)_68_	1540	9.06 × 10^5^	1620	1.9	1.7 × 10^4^	–	(181, 0.085)
Au_114_(PET)_80_	1732	7.28 × 10^5^	1870	1.0	6.1 × 10^3^	–	(206, 0.073)

A comparison of brightness as a function of emission wavelength (Chart 1, see Table S1 for details) illustrates that Au QRs outperform the state-of-the-art SWIR organic dyes by 2 orders of magnitude in the 1100–1400 nm region, meanwhile extending the emission range beyond the NIR-IIb (1500–1700 nm) and reaching 1870 nm (peak) or 2200 nm (edge), well beyond the emission range of reported dyes. We note that previously Meador et al. reported silicon-RosIndolizine (SiRos) dyes with emission peaks at 1557 and 1700 nm, but the brightness was only 3.1 M^–1^ cm^–1^ and 1.1 M^–1^ cm^–1^, respectively.^9^ Thus, in the region of >1500 nm, which is highly desirable for bioimaging and single quantum emitting applications, Au QRs are almost 4 orders of magnitude brighter than SiRos dyes and more than 2 orders of magnitude brighter than the very recently reported dye with a brightness of 61.3 M^–1^ cm^–1^ at 1650 nm.^6^ Other than organic dyes, we further benchmarked Au QRs against other representative SWIR-emitting material classes, including conjugated polymers, quantum dots, metal–organic frameworks, and atomically precise metal nanoclusters (Table S1), none of which simultaneously achieve the combination of ultrahigh brightness and continuously tunable SWIR emission demonstrated by Au QRs. This unprecedented brightness and spectral breadth establish Au QRs as a new benchmark platform for SWIR fluorescence and photonics.

**Chart 1:**
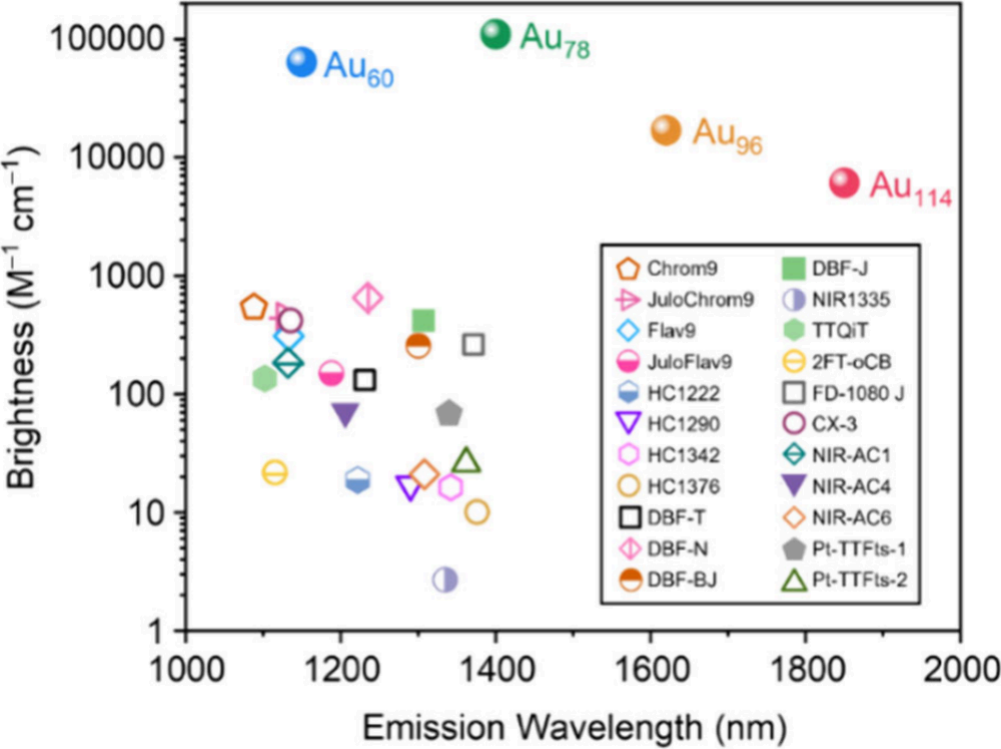
Comparison of Brightness between Au QRs and Representative SWIR Dyes (Details Listed in Table S1)

The Au QRs with SWIR emission in the 1000–2200 nm region are particularly advantageous for biomedical imaging, as this spectral region enables deep tissue penetration, reduced photon scattering, and minimal autofluorescence, which enable high-contrast, high-resolution imaging.^4–10,14,16^ Here, we transfer Au QRs into aqueous phase using an amphiphilic polymer (Pluronic F-127, abbreviated F-127) to wrap and stabilize Au QRs in water.^35^ As shown in Figure 3A, F-127 consists of hydrophilic poly­(ethylene oxide) (PEO) blocks at both ends and a hydrophobic poly­(propylene oxide) (PPO) block in the middle, whose self-assembly enables the encapsulation of Au QRs, forming stable and well-dispersed polymer-coated nanostructures in aqueous solution. We used Au_60_ to investigate the influence of F-127 wrapping on the PL properties; note that D_2_O was used as the solvent to inhibit H_2_O overtone vibrational reabsorption of PL (Figure S8A). As shown in Figure 3B, encapsulating Au_60_ with a low dose of F-127 (e.g., 5 mg) leads to a large reduction in PL intensity, with the QY dropping from 20.9% (pristine Au_60_ in toluene-*d*_8_, the pink star in Figure 3C) to 4.4%, which is caused by poor dispersion of Au QRs without sufficient F-127. As the amount of F-127 increases, the PL intensity of Au_60_@F-127 progressively recovers (Figure 3B) and the QY eventually reaches 17.3% (Figure 3C), comparable to that of pristine Au_60_. Longer Au QRs, including Au_78_, Au_96_, and Au_114_, can be similarly encapsulated in F-127 using the same approach (Figure S8B–D and Figure S9).

**Figure 3:**
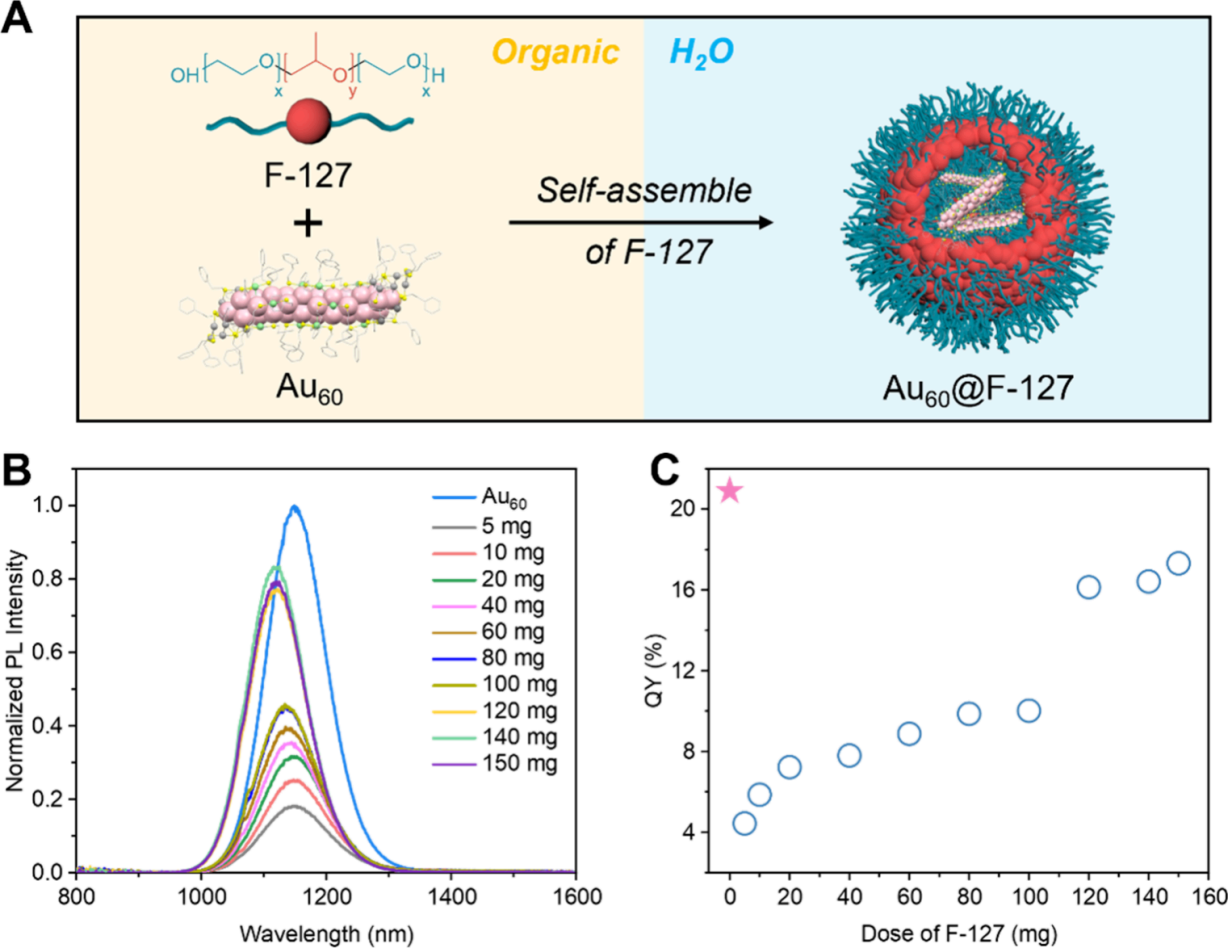
(A) Schematic illustration of the F-127 coating of Au_60_ to form water-dispersible Au_60_@F-127. (B) PL spectra of Au_60_ wrapped with varying doses of F-127. (C) QY of Au_60_ as a function of F-127 dose with the original QY in organic solvent marked by a pink star.

In summary, this work reports ultrabright and tunable SWIR fluorescence spanning 1000–2200 nm (edge-to-edge) from a periodic series of Au QRs with precisely controlled diameter and lengths. The strongly polarized excitons, together with rod-length-enabled suppression of ISC, leads to significantly enhanced fluorescence QYs. The emission brightness of these Au QRs is 2 orders of magnitude brighter than the state-of-the-art organic dyes in the 1100–1400 nm region. Meanwhile, Au QRs extend the emission range well beyond the NIR-IIb (1500–1700 nm) and reach 1870 nm (peak) or 2200 nm (edge) and are almost 4 orders of magnitude brighter than SiRos1557 and SiRos1700 dyes. The SWIR region of >1500 nm is highly desirable for high-resolution, deep tissue bioimaging and single quantum emitting applications. Collectively, the findings highlight the potential of atomically precise nanoclusters to expand the performance boundaries of SWIR fluorophores.

## Supplementary Material



## Notes

Details of the synthesis and characterization; supporting Figures S1–S9 (PDF)
