# Supplementary material for: Gold Quantum Rods Emit in the Shortwave Infrared (1000–2200 nm) with 104–5 M–1 cm–1 Ultrabrightness
Source: J Am Chem Soc. 2026 Mar 18;148(12):12520–5. doi: 10.1021/jacs.6c02971 (PMC13047534; doi:10.1021/jacs.6c02971)
Supplement: Supplementary file 1 [file ja6c02971_si_001.pdf]

Supporting Information:

Gold Quantum Rods Emit in the Shortwave Infrared (1000–2200 nm) with $10^{4\text{-}5}$ M^{-1} cm^{-1} Ultra-Brightness

Lianshun Luo, Guiying He, Zhongyu Liu, Avirup Sardar, Weijie Ji, Abhrojyoti Mazumder, Sihan Chen, Qi Li, and Rongchao Jin*

Department of Chemistry, Carnegie Mellon University, Pittsburgh PA 15213, United States.

*To whom correspondence should be addressed: rongchao@andrew.cmu.edu (R.J.)

Chemicals

Tetrachloroauric (III) acid ($HAuCl_4{\cdot}3H_2O$, 99.999% metal basis, Aldrich), phenylethyl mercaptan (PET, >99%, Aldrich), sodium borohydride (powder, ≥98%, Sigma), benzimidazole (98%, Aldrich), 2-bromopropane (99%, Aldrich), dimethyl sulfide (SMe_2, >99%, Aldrich), potassium carbonate (K_2CO_3, 99%, Aldrich), toluene-d8 (99 atom % D, Sigma), deuterium oxide (D_2O, 99.9 atom % D, Sigma), tetrachloroethylene (C_2Cl_4), methanol, ethanol, ethyl ether, ethyl acetate, pentane, dichloromethane (DCM), chloroform, *n*-hexane and acetonitrile (HPLC grade for all solvents) were used as received. Thin-layer chromatography (TLC) plates were from iChromatography (silica gel, 250 μm).

Synthesis of chloro(dimethylsulfide)gold(I) (AuCl(SMe$_2$))

$HAuCl_4{\cdot}3H_2O$ (500 mg, 1.27 mmol) was dissolved in ethanol (20 mL), followed by the addition of SMe_2 (280 μL, 3.81 mmol), and the solution was vigorously stirred for 2 hours. After that, the white precipitate was collected by centrifugation, which was washed with ethyl ether and finally dried to give the product as a white powder.

Synthesis of 1,3-diisopropylbenzimidazolium bromide (iPr$_2$-bimy·HBr)

Benzimidazole (1.18 g, 10 mmol) and K_2CO_3 (760 mg, 5.5 mmol) were added into acetonitrile (8 mL) and the mixture was rapidly stirred at ambient temperature for 1 hour. Following that, 2-bromopropane (5.4 mL, 57.5 mmol) was added to the suspension, and the reaction mixture was vigorously stirred under reflux conditions for 24 hours, followed by the addition of a second portion of 2-bromopropane (5.4 mL, 57.5 mmol). The reaction mixture was vigorously stirred under reflux for an additional 48 hours. After removing the solvent under reduced pressure, DCM was added to the residues, and the upper supernatant after centrifugation was collected. The solvent of the supernatant was removed under reduced pressure to produce a spongy solid, which was washed by ethyl acetate to afford the desired product as a white powder.

Synthesis of NHC-Au-Br complex ($^{i}Pr_2$-bimy·AuBr)

$^{i}Pr_2$-bimy·HBr (1337.4 mg, 4.725 mmol), AuCl(SMe_2) (1393.4 mg, 4.725 mmol), and K_2CO_3 (653.5 mg, 4.725 mmol) were added into acetone (20 mL) and the mixture was vigorously stirred

under reflux conditions for 2 hours. After that, the solvent in the suspension was removed under reduced pressure. DCM was added to the residues, and the upper supernatant after centrifugation was collected. The solvent of the supernatant was removed under reduced pressure to give the solid product, which was washed with pentane and finally dried to afford the desired product as a gray powder.

Synthesis of Au quantum rods

$^{i}Pr_2$-bimy·AuBr (120 mg, 0.25 mmol) and PET (67 μL, 0.5 mmol) were dissolved in a mixture of chloroform (15 mL) and ethanol (5 mL). The mixture gradually turned cloudy white (Au^{I}-PET formed). After 1 hour of stirring, the suspension was reduced to nanoclusters (NCs) by the addition of sodium borohydride (95 mg, 2.5 mmol, dissolved in 5 mL ice-cold H_2O) under darkness. A black solution was formed. The reaction was allowed to continue for 6 hours, and then the solvent was removed under reduced pressure. The mixture of Au NCs was thoroughly washed with methanol, extracted with DCM, and concentrated for TLC separation. The mixture of Au NCs was pipetted onto a TLC plate, and the separation was conducted in a developing tank (developing solvent 1:1 (v/v) DCM:*n*-hexane). Four bands corresponding to $Au_{60}(PET)_{44}$, $Au_{78}(PET)_{56}$, $Au_{96}(PET)_{68}$, and $Au_{114}(PET)_{80}$ were cut off (**Figure S1**) and dissolved in DCM for characterization.

Plate-shaped crystals of $Au_{60}(PET)_{44}$ were obtained via evaporation of acetonitrile into a toluene solution of the nanoclusters at room temperature for two weeks.

Organic-to-Aqueous Phase Transfer of Au QRs using F-127

A stock solution (3 mg/mL) of Au_{60} in THF was first prepared. Then, 50 μL of this stock was mixed with F-127, which had been dissolved in 150 μL of THF at varying masses. The mixture was sonicated for a few minutes to ensure thorough mixing. Subsequently, 5 mL of D_2O was added, followed by another round of sonication to achieve uniform dispersion. The slight blue shift of PL observed upon F-127 encapsulation (**Figure 3A)** is attributed to the change in local dielectric microenvironments[s48,s49] of Au_{60} QR. The irregularity between 100 and 120 mg of F-127 may be due to the threshold for the encapsulation, at which the F-127 polymer coverage becomes sufficient to fully disperse the Au_{60} QR; consequently, the PL efficiency nearly recovers.

For Au_{78}, Au_{96}, Au_{114}, separate 3 mg/mL stock solutions in THF were prepared. Each solution (50 μL) was mixed with 150 mg of F-127 dissolved in 150 μL of THF. After sonication to ensure thorough mixing, 5 mL of D_2O was added, and the mixtures were sonicated again to achieve uniform aqueous dispersions. After F-127 encapsulation, the QYs of Au_{78} and Au_{96} decreased to 9.6% and 0.7%, respectively (**Figure S8**), and Au_{114} emission is almost overwhelmed by D_2O vibrational overtone absorption (which becomes significant > 1700 nm, **Figure S9** (all were 1 cm beam path); ultrathin cells should lessen the re-absorption).

UV-Vis-NIR measurements

UV-Vis-NIR spectra of the nanoclusters were collected with a UV-3600 Plus UV-VIS-NIR spectrophotometer (Shimadzu, detection range: 185 to 3300 nm).

Steady-state photoluminescence and lifetime measurements

Steady-state photoluminescence (PL) spectra were recorded using an FLS–1000 spectrofluorometer (Edinburgh Instruments). Emission below 1650 nm was measured using a wide-range photomultiplier tube (PMT–1700, cooled to –80 °C with liquid nitrogen). For SWIR emission above 1650 nm, an InAs photovoltaic detector with lock-in amplification was used (detection to ~3,000 nm).

The parameters for PL measurements of Au_{60}, Au_{78}, Au_{60}@F-127 and Au_{78}@F-127: both excitation and emission slit bandwidths were set to 10 nm, and measurements used a PMT-1700 detector. The PL measurements of Au_{96}, Au_{114}, Au_{98}@F-127 and Au_{114}@F-127 used an InAs photovoltaic detector up to ~3000 nm, with the excitation and emission slit bandwidths set to 15 and 40 nm, respectively, and a dwell time of 0.2 s for 10 runs.

The PL lifetimes were measured by time-correlated single photon counting (TCSPC) with a PMT-1700 (up to 1650 nm only) on the same instrument.

Quantum yield measurement

The absolute QYs (PL up to 1650 nm with a PMT-1700 detector) were measured using an integrating sphere, the standard sample holder was removed and replaced with the integrating sphere. First, a blank spectrum was recorded by placing a cuvette containing only the solvent in the integrating sphere and performing five repeated emission scans, starting from 20 nm longer than the excitation wavelength to the longest wavelength of the detector. Next, the blank was replaced with a dilute solution of Au QRs adjusted to an OD of 0.1 at the excitation wavelength. The emission scan was then repeated five times over the same spectral range. PLQY was determined using the software of FLS-1000.

The PLQYs of Au_{96} and Au_{114} were measured by an InAs photovoltaic detector (up to ~3000 nm) using Au_{78} as a reference (note: the InAs detector is not compatible with the integrating sphere method). For this relative method, the PL intensity of Au_{78} was also measured by the InAs photovoltaic detector under the same parameters to serve as a reference for determining the QYs of Au_{96} and Au_{114} (Figure S2).

The relative quantum yields (Φ_S) of other samples (denoted S in the equation below) beyond the coverage by the PMT-1700 detector were measured by using a reference standard (denoted R in the equation) under the same instrumental settings and calculated using the following equation:

$$\Phi_S = \Phi_R\left(\frac{I_S}{I_R}\right)\left(\frac{1-10^{-A_R}}{1-10^{-A_S}}\right)\left(\frac{n_S}{n_R}\right)^2$$

where, Φ_R is the QY of the reference standard, I is the integrated PL intensity (for S or R), A is the absorbance of the solution at the excitation wavelength (for S and R), and n is the refractive index of the solution solvent.

Supporting figures

(Figs S1-S9, see next pages)

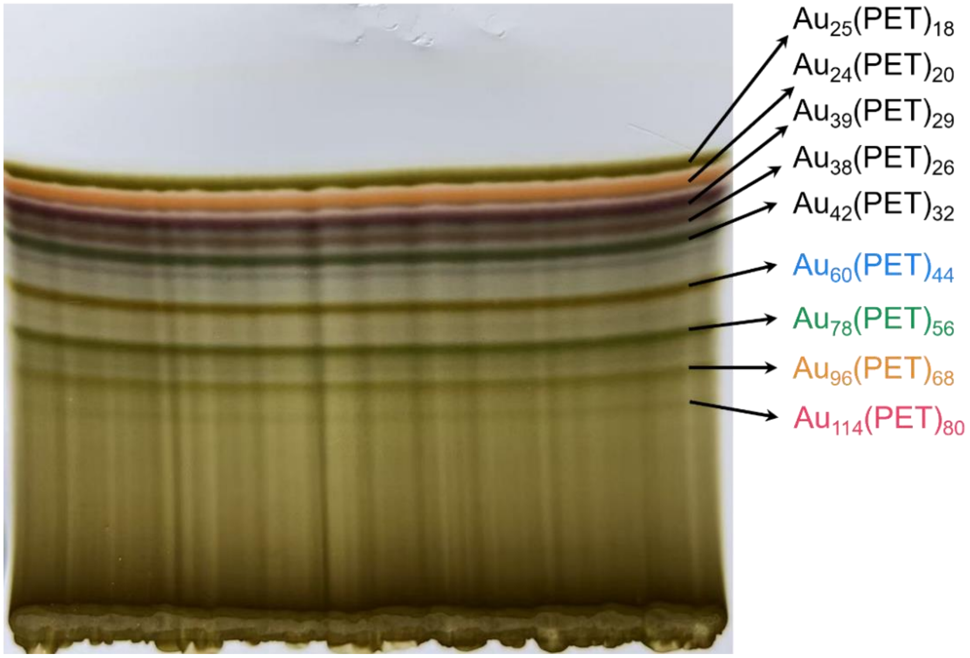

Figure S1. Thin-layer chromatography separation of $Au_{60}(PET)_{44}$, $Au_{78}(PET)_{56}$, $Au_{96}(PET)_{68}$, and $Au_{114}(PET)_{80}$ from the product mixture.

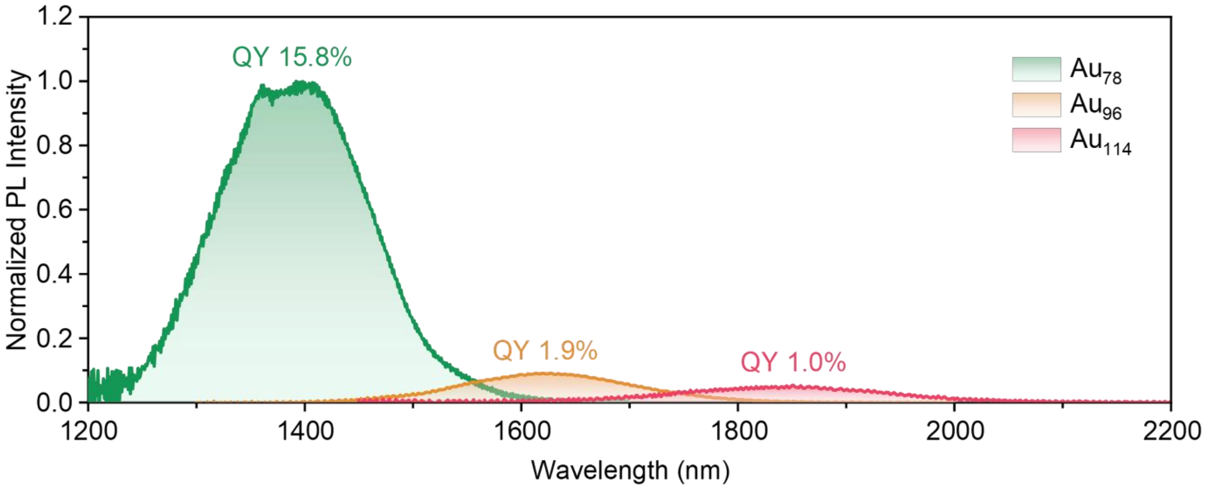

Figure S2. Normalized PL spectra of Au_{78}, Au_{96}, and Au_{114} in toluene-d_8. The excitation wavelengths used for the PL measurements were 1320, 1540, and 1700 nm for Au_{78}, Au_{96}, and Au_{114}, respectively. For PL measurements (Au_{78} as the reference for Au_{96} and Au_{114} QY measurements), an InAs photovoltaic detector (up to ~3000 nm) was used; excitation and emission slit bandwidths were set to 12.7 and 45 nm, respectively, with a dwell time of 0.2 s for 20 runs.

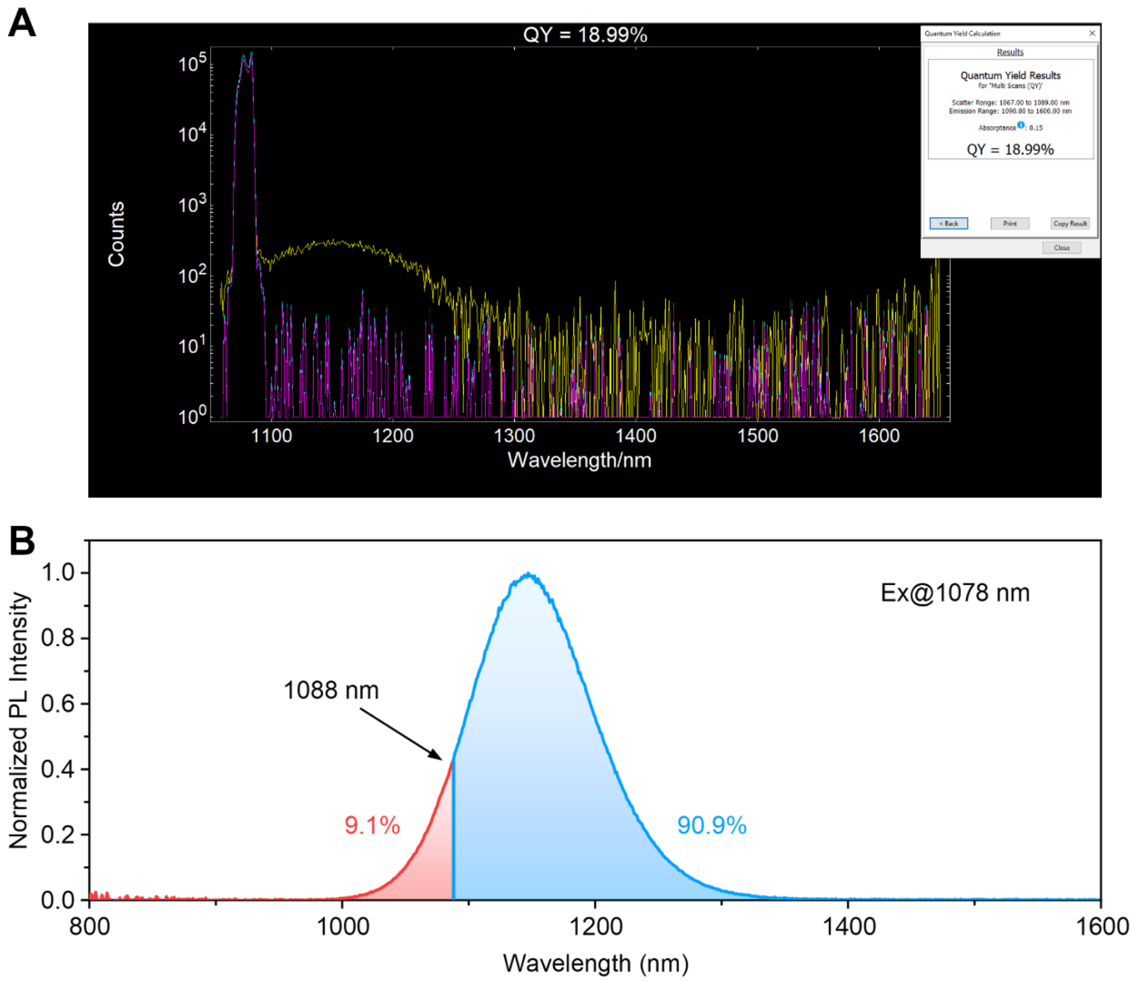

Figure S3. (A) QY measurement of Au_{60} in toluene-d_8 under the excitation of 1078 nm, corresponding to the SWIR absorption peak of Au_{60}. Excitation and emission slit bandwidths were set to 10 nm and 0.95 nm, respectively. (B) PL spectrum of Au_{60} in toluene-d_8 under 1078 nm excitation, showing the integrated PL intensity contributions from 800-1088 nm (9.1% of the total PL intensity, i.e. area) and 1088-1600 nm (90.9% of the total PL intensity).

The excitation wavelength was set to 1078 nm with an excitation slit bandwidth of 10 nm. As a result, the system integrates the emission signal from 1088 to 1600 nm, excluding the portion between 800 and 1088 nm. Therefore, the measured QY value (18.99% in **Figure S3A**) represents only the emission >1088 nm. To obtain the accurate total QY of Au_{60} under 1078 nm excitation, the emission between 800 and 1088 nm should be included (9.1% of the total PL intensity in **Figure S3B**), yielding a corrected QY of $\frac{1}{90.9\%} * 18.99\% = \mathbf{20.9\%}$.

Note: the 400 nm excited PL peak and profile remain unchanged, but the QY is lower.

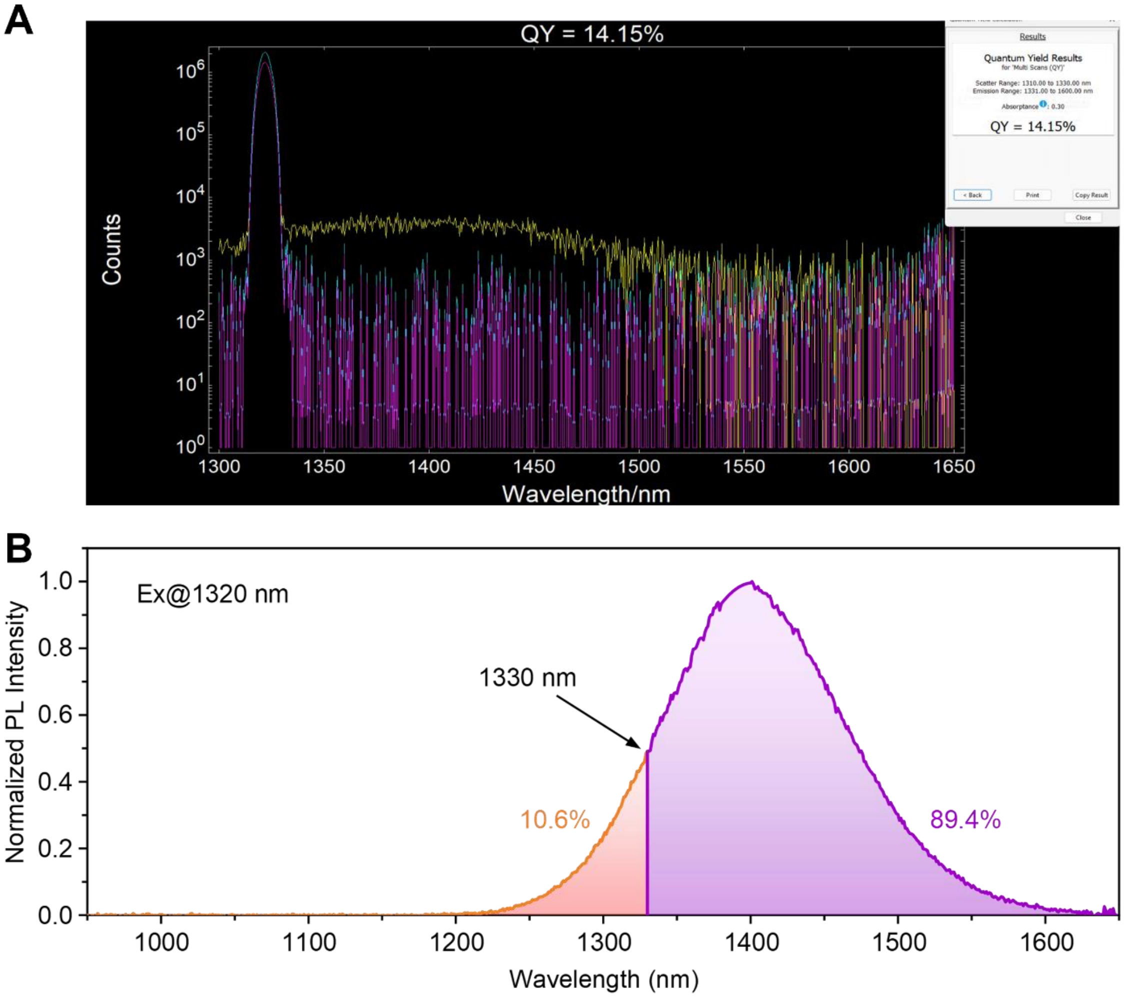

Figure S4. (A) QY measurement of Au_{78} in toluene-d_8 under the excitation of 1320 nm, corresponding to the SWIR absorption peak of Au_{78}. Excitation and emission slit bandwidths were set to 10 nm and 1.5 nm, respectively. (B) PL spectrum of Au_{78} in toluene-d_8 under 1320 nm excitation, showing the integrated PL intensity contributions from 900-1330 nm (14.15% of the total PL intensity) and 1330-1650 nm (89.4% of the total PL intensity).

The excitation wavelength was set to 1320 nm with an excitation slit bandwidth of 10 nm. As a result, the system integrates the emission signal from 1330 to 1650 nm, excluding the portion between 900 and 1330 nm. Therefore, the measured QY value (10.19% in **Figure S4A**) represents only the emission beyond 1330 nm. To obtain the accurate total QY of Au_{78} under 1320 nm excitation, the emission between 900 and 1330 nm must also be included (89.4% of the total PL intensity in **Figure S4B**), yielding a corrected QY of $\frac{1}{89.4\%} * 14.15\% = \mathbf{15.8}\%$.

Note: the 400 nm excited PL peak and profile remain unchanged, but the QY is lower.

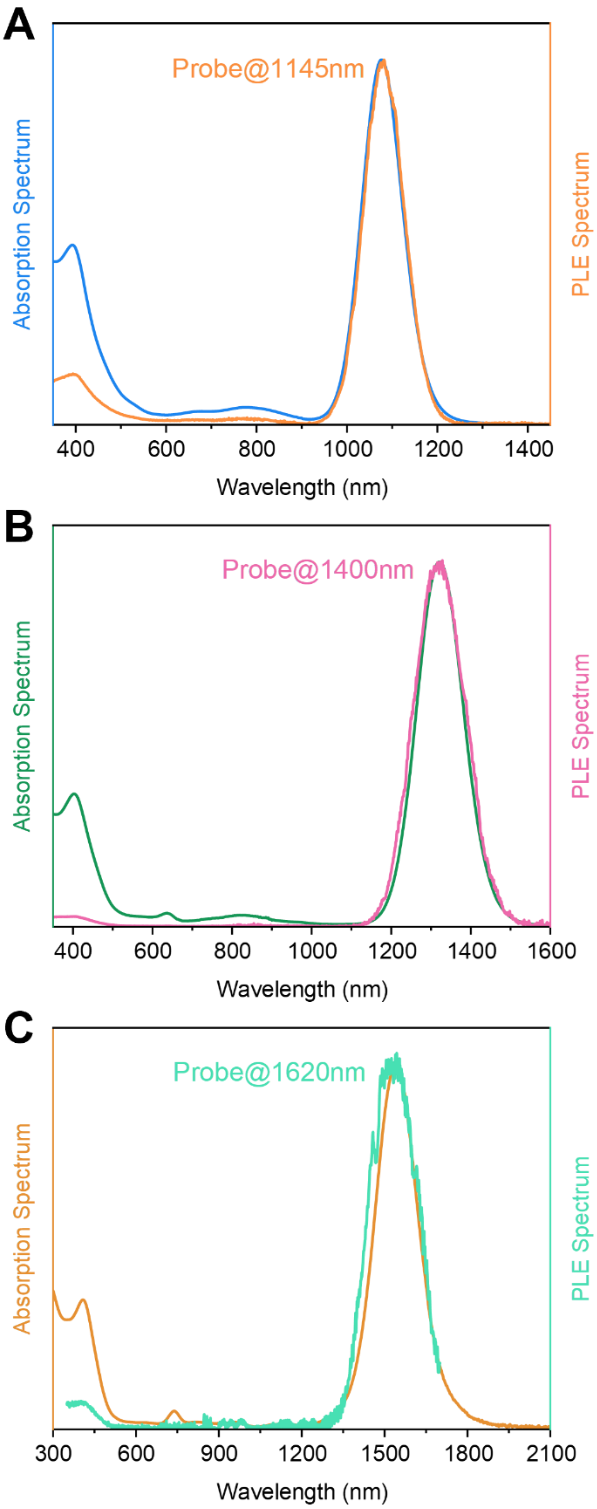

Figure S5. Comparison of the optical absorption spectra and excitation spectra of (A) Au_{60}, (B) Au_{78}, and (C) Au_{96}. Note: the PLE of Au_{114} was out of the range of detection, hence, not measured.

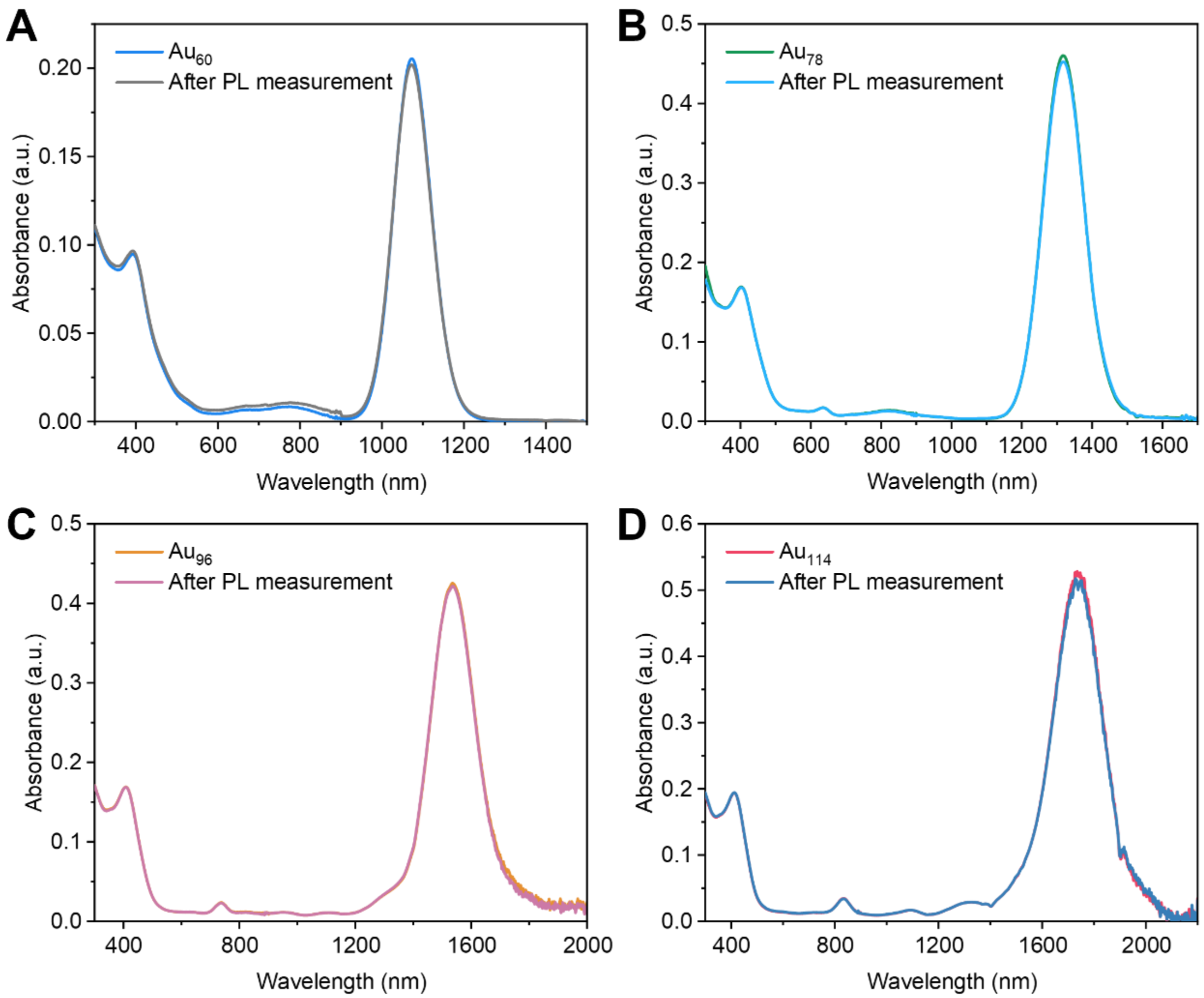

Figure S6. Photostability of (A) Au_{60}, (B) Au_{78}, (C) Au_{96}, and (D) Au_{114}, assessed by comparing their optical absorption spectra before and after PL measurements. All QRs were dissolved in toluene-d_8. No spectral degradation was observed after measurements.

Note: These QRs are also stable in solid state (for long term storage) and can be re-dispersed.

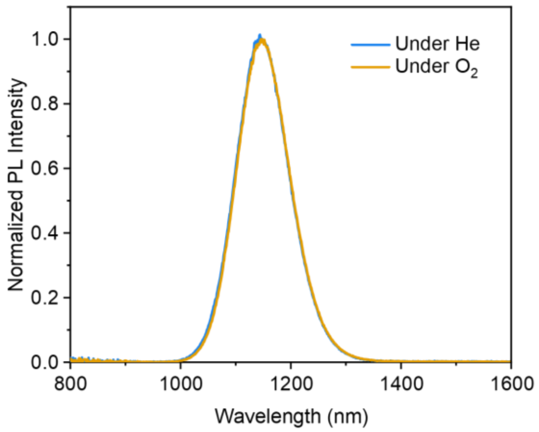

Figure S7. PL spectra of Au_{60} in toluene-d_8 under He and O_2 atmospheres. Excitation = 1078 nm, both excitation and emission slit widths = 10 nm.

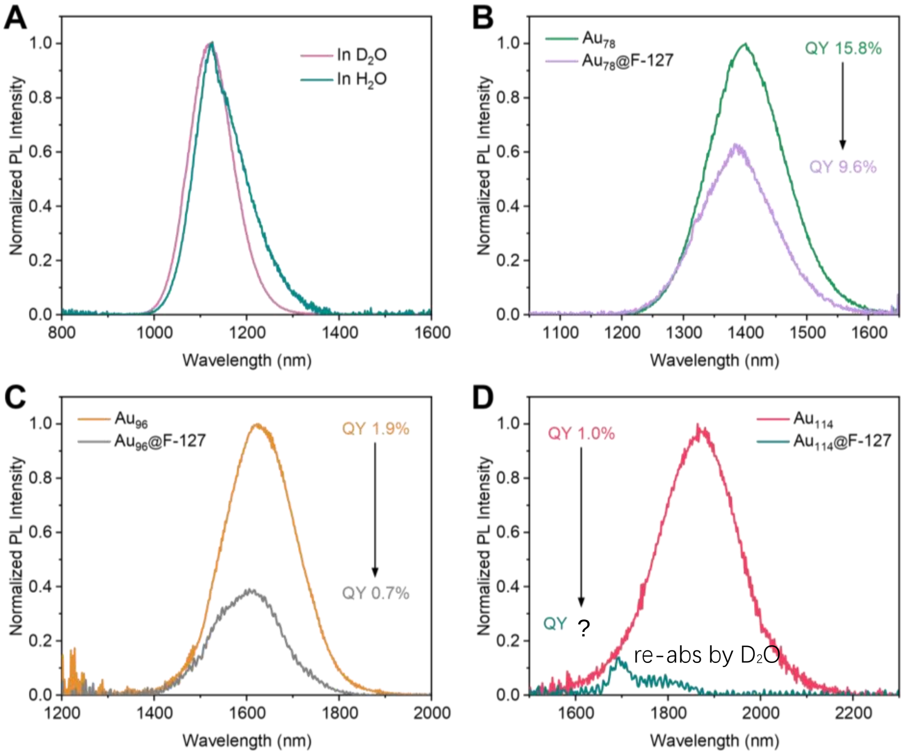

Figure S8. (A) PL spectra of Au_{60}@F-127 in D_2O and H_2O. (B) PL spectra and QYs of Au_{78} in toluene-d_8 and Au_{78}@F-127 in D_2O. (C) PL spectra and QYs of Au_{96} in toluene-d_8 and Au_{96}@F-127 in D_2O. (D) PL spectra of Au_{114} in toluene-d_8 and Au_{114}@F-127 in D_2O (note: severe re-absorption at >1700 nm by D_2O (1 cm path, see Figure S9); ultrathin cells should lessen the re-absorption).

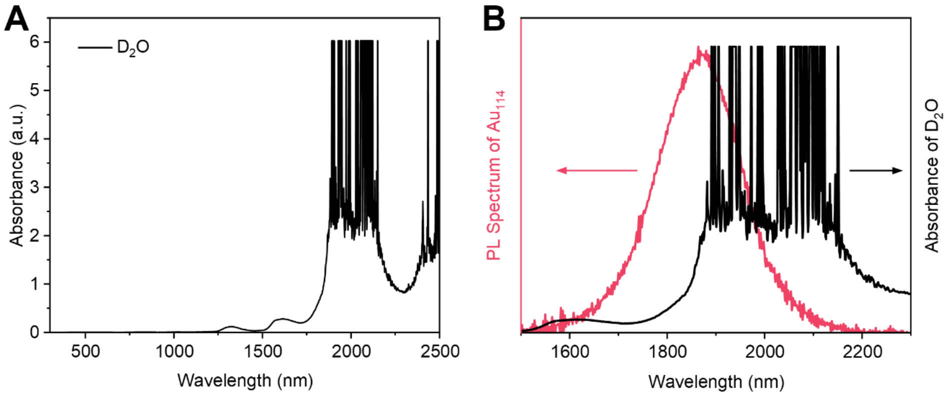

Figure S9. (A) Optical absorption spectrum of D_2O. (B) Comparison of PL spectrum of Au_{114} in toluene-d_8 and absorption spectrum of D_2O (1 cm path; ultrathin cells should lessen the reabsorption).

Table S1. Comparison of Au QRs with Representative SWIR Fluorophores across Major Material Classes.

Class	Representative Materials	Em. peak (nm)	QY (%)	Brightness (M^{-1} cm^{-1})	Ref.
Au QRs	Au_{60}	1145	20.9	6.2×10^4	This work
	Au_{78}	1400	15.8	1.1×10^5	
	Au_{96}	1620	1.9	1.7×10^4	
	Au_{114}	1870	1.0	6.1×10^3	
Other Au NCs	$Au_{25}(SG)_{18}$	1120	~0.13	–	1,2
	$Au_{24}Cd_1MPA$	1020	1.1	–	3
	$Au_{44}MBA_{26}$	1080/1280	4.5	–	4
	$Au_{76}(p\text{-MBT})_{42}$	970	30	–	5
	$Au_{76}(p\text{-MBA})_{44}$	~1060	60	–	6
	$Au_{22}Cu_1$	926/1020	0.16	–	7
	$Au_{37}(TBBT)_{21}(TPP)_2$	1152	1.5	–	8
	$Au_{42}(PET)_{32}$	875/1045	18	–	9
	$Au_{66}(PET)_{38}$	1050	~0.67	–	10
Organic dyes and organometallic complexes	Chrom9	1088	0.5	540	11
	JuloChrom9	1128	0.31	450	11
	Flav9	1133	0.19	310	11
	JuloFlav9	1188	0.15	150	11
	HC1222	1222	0.016	18.8	12
	HC1290	1290	0.010	17.2	12
	HC1342	1342	0.015	16.3	12
	HC1376	1376	0.011	10.1	12
	DBF-T	1230	0.116	135	13
	DBF-N	1235	0.377	652	13
	DBF-BJ	1300	0.090	257	13

	DBF-J	1305	0.136	430	13
	NIR1335	1335	0.006	2.7	13
	TTQiT	1102	0.37	135.42	14
	2FT-oCB	1115	0.095	21.85	15
	FD-1080 J	1370	0.54	270	16
	CX-3	1135	0.82	420	17
	NIR-AC1	1132	0.136	184	18
	NIR-AC4	1206	0.063	71	18
	NIR-AC6	1308	0.024	21.5	18
	Pt-TTFts-1	1340	0.065	68.5	19
	Pt-TTFts-2	1362	0.028	26.0	19
Semiconducting polymers	m-PBTQ4F NPs	1089	7.1	–	20
	TT-3T CPs	1070	1.75	–	21
	P1 NPs	1257	0.1	–	22
	Dual-emission Pdots	1100	1.9	–	23
	TTQ-2TC NPs	1270	0.03	–	24
	PFTQ-PEG-Gd NPs	1056	0.38	–	25
	Ptcc-SeBTa-NIR1380 NPs	1300	0.05	–	26
	P1-Pdots	1095	1.0	–	27
MOFs	SION-100-NH_2	1025	1.26	–	28
	$[Nd(tfbdc)_{0.5}(bta)_2(CH_3OH)_2]\cdot CH_3OH$	900/1065/1345	4.2	–	29
	$[Nd_2(L^a)_2(DMAC)_2]_n\cdot nH2O$	1054	3.5	–	30
	$[Nd_5L^b{}_4(NO_3)_4(OH)_2]\cdot OH$	882/1067/1335	0.12	–	31
	$[Pr(TFBDC)_{1.5}(H_2O)]\cdot 2H_2O$	1020/1225	5	–	32
	$[Sm_2(HPI_2C)_3(DMF)_2(H_2O)_2]\cdot 2DMF$	890/940/1020/1170	0.37	–	33
	Nd-MOF@Yb-MOF@SiO_2@Fe_3O_4	890/978/1056	0.07	–	34
Quantum Dots	Ag/Ag_2S JNPs	1250	4.91	–	35
	PEG-Ag_2S NPs	1250	2.3	1.03×10^4	36

	GSH-Ag_2Se	1100	5	–	37
	$Ag_{2-3x}Bi_xS$	1036	1	–	38
	$Pb_{1-x}Cd_xS$	1100	17.72	–	39
	$Ag_2Te@Ag_2S$	1560	4.3	–	40
D-A structure small molecules	BTFQ/DMPC	1113	0.63	~65	41
	NK 1143-SC12 NPs	1143	0.16	113	42
	2TT-oC26B NPs	1034	11.5	–	43
	PCP-BDP2 NPs	1010	6.4	–	44
	TTQiT NPs	1102	3.7	2855	45
	FT-TQT	1034	0.49	–	46
	CTB1125-NPs	900-1400	4.84	–	47

Supporting references:

1. Liu, H.; Hong, G.; Luo, Z.; Chen, J.; Chang, J.; Gong, M.; He, H.; Yang, J.; Yuan, X.; Li, L.; Mu, X.; Wang, J.; Mi, W.; Luo, J.; Xie, J.; Zhang, X.-D. Atomic-Precision Gold Clusters for NIR-II Imaging. *Adv. Mater.* **2019**, *31*, 1901015.
2. Liu, P. F.; Shi, T. Z.; Li, H. W.; Chen, H. Y.; Huang, Y.; Ma, H. Z.; Zhu, T. Y.; Zhao, R.; Li, Y.; Xin, Q. Airy beam assisted NIR-II light-sheet microscopy. *Nano Today.* **2022**, *47*, 101628.
3. Huang, Y.; Chen, K.; Liu, L.; Ma, H. Z.; Zhang, X. N.; Tan, K. X.; Li, Y.; Liu, Y.; Liu, C. L.; Wang, H.; Zhang, X. D. Single Atom-Engineered NIR-II Gold Clusters with Ultrahigh Brightness and Stability for Acute Kidney Injury. *Small* **2023**, *19*, 2300145.
4. Yang, G.; Pan, X.; Feng, W.; Yao, Q.; Jiang, F.; Du, F.; Zhou, X.; Xie, J.; Yuan, X. Engineering Au_{44} Nanoclusters for NIR-II Luminescence Imaging-Guided Photoactivatable Cancer Immunotherapy. *ACS Nano* **2023**, *17*, 15605-15614.
5. Wang, Y.; Sardar, A.; Liu, Z.; Gianopoulos, C. G.; He, G.; Liu, X.; Chen, S.; Kirschbaum, K.; Mazumder, A.; Cotlet, M.; Jiang, D. E.; Jin, R., $Au_{76}(SC_6H_4$-*p*-$CH_3)_{42}$ Square Quantum Platelet: One-Dimensional Growth of Quantum Rods Turns 90 Degrees. *J. Am. Chem. Soc.* **2025**, *147*, 42752-42757.
6. Luo, X.; Xiao, H.; He, S.; Zhao, T.; Zhang, G.; Liu, J., Photooxidation-Induced Ultrabright and Ultraphotostable NIR-II Emissive Water-Soluble Gold Nanoclusters. *Angew. Chem. Int. Ed.* **2025**, *64*, e202511751.
7. Ma, H.; Zhang, X.; Liu, L.; Huang, Y.; Sun, S.; Chen, K.; Xin, Q.; Liu, P.; Yan, Y.; Wang, Y. Bioactive NIR-II gold clusters for three-dimensional imaging and acute inflammation inhibition. *Sci. Adv.* **2023**, *9*, eadh7828.
8. Tan, Y.; Li, K.; Xu, J.; Li, Q.; Yang, S.; Chai, J.; Pei, Y.; Jia, D.; Zhu, M. A single-gold-atom addition regulates sharp redshift in the fluorescence of atomically precise nanoclusters. *Nanoscale* **2024**, *16*, 15663-15669.
9. Luo, L.; Liu, Z.; Mazumder, A.; Jin, R. Raising near-infrared photoluminescence quantum yield of Au_{42} quantum rod to 50% in solutions and 75% in films. *J. Am. Chem. Soc.* **2024**, *146*, 27993-27997.
10. Gu, W.; Zhou, Y.; Wang, W.; You, Q.; Fan, W.; Zhao, Y.; Bian, G.; Wang, R.; Fang, L.; Yan, N.; Xia, N.; Liao, L.; Wu, Z. Concomitant Near-Infrared Photothermy and Photoluminescence of Rod-Shaped $Au_{52}(PET)_{32}$ and $Au_{66}(PET)_{38}$ Synthesized Concurrently. *Angew. Chem., Int. Ed.* **2024**, *63*, e202407518.
11. Spearman, A. L.; Lin, E. Y.; Mobley, E. B.; Chmyrov, A.; Arús, B. A.; Turner, D. W.; Garcia, C. A.; Bui, K.; Rowlands, C.; Bruns, O. T. High-Resolution Multicolor Shortwave Infrared Dynamic Imaging with Chromenylium Nonamethine Dyes. *J. Am. Chem. Soc.* 2025, 147, 17384-17393.
12. Yang, Y.; Sun, C.; Wang, S.; Yan, K.; Zhao, M.; Wu, B.; Zhang, F. Counterion-Paired Bright Heptamethine Fluorophores with NIR-II Excitation and Emission Enable Multiplexed Biomedical Imaging. *Angew. Chem., Int. Ed.* **2022**, *61*, e202117436.
13. Chowdhury, P.; Lu, Z.; Su, S.; Liu, M.; Lin, C.; Wang, M.; Luo, Y.; Lee, Y.; Chiang, H.

K.; Chan, Y. Ultrabright Dibenzofluoran-Based Polymer Dots with NIR-IIa Emission Maxima and Unusual Large Stokes Shifts for 3D Rotational Stereo Imaging. *Adv. Healthc. Mater.* **2024**, *13*, 2400606.

14. Li, Y.; Zha, M.; Kang, T.; Li, C.; Wu, X.; Wang, S.; Lu, S. B.; Lee, Y. S.; Wu, Y. R.; Ni, J. S.; Li, K. Promoted NIR-II Fluorescence by Heteroatom-Inserted Rigid-Planar Cores for Monitoring Cell Therapy of Acute Lung Injury. *Small* **2022**, *18*, e2105362.
15. Feng, Z.; Li, Y.; Chen, S.; Li, J.; Wu, T.; Ying, Y.; Zheng, J.; Zhang, Y.; Zhang, J.; Fan, X.; Yu, X.; Zhang, D.; Tang, B. Z.; Qian, J. Engineered NIR-II Fluorophores with Ultralong-Distance Molecular Packing for High-Contrast Deep Lesion Identification. *Nat. Commun.* **2023**, *14*, 5017.
16. Sun, C.; Li, B.; Zhao, M.; Wang, S.; Lei, Z.; Lu, L.; Zhang, H.; Feng, L.; Dou, C.; Yin, D.J-Aggregates of Cyanine Dye for NIR-II in Vivo Dynamic Vascular Imaging Beyond 1500 nm. *J. Am. Chem. Soc.* **2019**, *141*, 19221-19225.
17. Lei, Z.; Sun, C.; Pei, P.; Wang, S.; Li, D.; Zhang, X.; Zhang, F. Stable, wavelength-tunable fluorescent dyes in the NIR-II region for *in vivo* high-contrast bioimaging and multiplexed biosensing. *Angew. Chem., Int. Ed.* **2019**, *58* (24), 8166-8171.
18. Ou, Y. F.; Xiang, H. Y.; Yang, X.; Wang, R. X.; Huan, S. Y.; Yuan, L.; Ren, T. B.; Zhang, X. B. Constructing stable and wavelength-extended heptamethine cyanines via donor ectopic substitution for NIR-IIa/b bioimaging. *Angew. Chem., Int. Ed.* **2025**, *64*, e202423978.
19. McNamara, L. E.; Zhou, A.; Anferov, S. W.; Krzyaniak, M.; Wasielewski, M.; Sun, L.; Boyn, J.-N.; Schaller, R. D.; Anderson, J. S. Above the energy gap law: Heavy chalcogenide substitution in NIR II-emissive diradicaloid qubits. *ChemRxiv*. Preprint, 2025. DOI: 10.26434/chemrxiv-2025-9xk7r.
20. Liu, Y.; Liu, J.; Chen, D.; Wang, X.; Zhang, Z.; Yang, Y.; Jiang, L.; Qi, W.; Ye, Z.; He, S. Fluorination Enhances NIR-II Fluorescence of Polymer Dots for Quantitative Brain Tumor Imaging. *Angew. Chem., Int. Ed.* **2020**, *59*, 21049-21057.
21. Zhou, H.; Lu, Z.; Zhang, Y.; Li, M.; Xue, D.; Zhang, D.; Liu, J.; Li, L.; Qian, J.; Huang, W. Simultaneous Enhancement of the Long-Wavelength NIR-II Brightness and Photothermal Performance of Semiconducting Polymer Nanoparticles. *ACS Appl. Mater. Interfaces* **2022**, *14*, 8705-8717.
22. Chen, Y.; Sun, B.; Jiang, X.; Yuan, Z.; Chen, S.; Sun, P.; Fan, Q.; Huang, W. Double-Acceptor Conjugated Polymers for NIR-II Fluorescence Imaging and NIR-II Photothermal Therapy Applications. *J. Mater. Chem. B* **2021**, *9*, 1002-1008.
23. He, S.; Chen, S.; Li, D.; Wu, Y.; Zhang, X.; Liu, J.; Song, J.; Liu, L.; Qu, J.; Cheng, Z. High Affinity to Skeleton Rare Earth Doped Nanoparticles for Near-Infrared II Imaging. *Nano Lett.* **2019**, *19*, 2985-2992.
24. Song, X.; Lu, X.; Sun, B.; Zhang, H.; Sun, P.; Miao, H.; Fan, Q.; Huang, W. Conjugated Polymer Nanoparticles with Absorption beyond 1000 nm for NIR-II Fluorescence Imaging System Guided NIR-II Photothermal Therapy. *ACS Appl. Polym. Mater.* **2020**, *2*, 4171-4179.
25. Hu, X.; Tang, Y.; Hu, Y.; Lu, F.; Lu, X.; Wang, Y.; Li, J.; Li, Y.; Ji, Y.; Wang, W.; Ye, D.; Fan, Q.; Huang, W. Gadolinium-Chelated Conjugated Polymer-Based Nanotheranostics for

Photoacoustic/Magnetic Resonance/NIR-II Fluorescence Imaging-Guided Cancer Photothermal Therapy. *Theranostics* **2019**, *9*, 4168-4181.
26. Liu, M. H.; Zhang, Z.; Yang, Y. C.; Chan, Y. H. Polymethine-Based Semiconducting Polymer Dots with Narrow-Band Emission and Absorption/Emission Maxima at NIR-II for Bioimaging. *Angew. Chem., Int. Ed.* **2021**, *60*, 983-989.
27. Liu, Y.; Liu, J.; Chen, D.; Wang, X.; Liu, Z.; Liu, H.; Jiang, L.; Wu, C.; Zou, Y. Quinoxaline-Based Semiconducting Polymer Dots for in Vivo NIR-II Fluorescence Imaging. *Macromolecules* **2019**, *52*, 5735-5740.
28. Nguyen, T. N.; Capano, G.; Gładysiak, A.; Ebrahim, F. M.; Eliseeva, S. V.; Chidambaram, A.; Valizadeh, B.; Petoud, S.; Smit, B.; Stylianou, K. C. Lanthanide-Based Near-Infrared Emitting Metal-Organic Frameworks with Tunable Excitation Wavelengths and High Quantum Yields. *Chem. Commun.* **2018**, *54*, 13271-13274.
29. Yao, X.; Wang, X.; Han, Y.; Yan, P.; Li, Y.; Li, G. Structure, Color-Tunable Luminescence, and UV-Vis/NIR Benzaldehyde Detection of Lanthanide Coordination Polymers Based on Two Fluorinated Ligands. *CrystEngComm* **2018**, *20*, 3335-3343.
30. Wang, X.; Yan, P.; Li, Y.; An, G.; Yao, X.; Li, G. Highly efficient white-light emission and UV-visible/NIR luminescence sensing of lanthanide metal-organic frameworks. *Cryst. Growth Des.* **2017**, *17*, 2178-2185.
31. Niu, M.; Leng, X.; Yang, X.; Schipper, D., A NIR luminescent "tetra-decker" Nd(III) salen nanocluster for rapid ratiometric fluorescence detection of quercetin. *J. Lumin.* **2022**, *250*, 119067.
32. Han, Y. Q.; Yan, P. F.; Sun, J. W.; An, G. H.; Yao, X.; Li, Y. X.; Li, G. M. Luminescence and white-light emitting luminescent sensor of tetrafluoroterephthalatelanthanide metal-organic frameworks. *Dalton Trans.* **2017**, *46*, 4642-4653.
33. Chen, L.; Yan, C.; Pan, M.; Wang, H. P.; Fan, Y. N.; Su, C. Y. Multi-mode white light emission in a Zn^{II} coordination polymer from excited-state intramolecular proton transfer (ESIPT) ligands. *Eur. J. Inorg. Chem.* **2016**, *2016*, 2676-2680.
34. Jiang, Y.-P.; Fang, X.-H.; Wang, Q.; Huo, J.-Z.; Liu, Y.-Y.; Wang, X.-R.; Ding, B. Near-infrared magnetic core-shell nanoparticles based on lanthanide metal-organic frameworks as a ratiometric felodipine sensing platform. *Commun. Chem.* **2023**, *6*, 96.
35. Zhang, X.; Wang, W.; Su, L.; Ge, X.; Ye, J.; Zhao, C.; He, Y.; Yang, H.; Song, J.; Duan, H. Plasmonic-Fluorescent Janus Ag/Ag_2S Nanoparticles for In Situ H_2O_2-Activated NIR-II Fluorescence Imaging. *Nano Lett.* **2021**, *21*, 2625-2633.
36. Shen, Y. L.; Lifante, J.; Zabala-Gutierrez, I.; de la Fuente-Fernandez, M.; Granado, M.; Fernandez, N.; Rubio-Retama, J.; Jaque, D.; Marin, R.; Ximendes, E.; Benayas, A. Reliable and Remote Monitoring of Absolute Temperature during Liver Inflammation *via* Luminescence-Lifetime-Based Nanothermometry. *Adv. Mater.* **2022**, *34*, 2107764.
37. Yang, L. L.; Zhao, W.; Liu, Z. Y.; Ren, M. T.; Kong, J.; Zong, X.; Luo, M. Y.; Tang, B.; Xie, J. H. Y.; Pang, D. W.; Liu, A. A. Acid-Resistant Near-Infrared II Ag_2Se Quantum Dots for Gastrointestinal Imaging. *Anal. Chem.* **2023**, *95*, 15540-15548.

38. Cui, D.; Han, L.; Jiang, W.; Chen, L.; Niu, N. $Ag_{2-3x}Bi_xS$ Quantum Dots as Single-Component Theranostic Agents for Second Near-Infrared Fluorescence Imaging-Guided Photothermal Therapy. *ACS Appl. Nano Mater.* **2023**, *6*, 1303-1314.
39. Zhang, H.; Sun, C.; Sun, L.; Xu, W.; Wu, W.; Chen, J.; Wang, B.; Yu, J.; Cui, P.; Zhang, F.; Tang, Y. Stable Monodisperse $Pb_{1-x}Cd_xS$ Quantum Dots for NIR-II Bioimaging by Aqueous Coprecipitation of Bimetallic Clusters. *Angew. Chem., Int. Ed.* **2022**, *61*, 2203851.
40. Zhang, Y.; Yang, H.; An, X.; Wang, Z.; Yang, X.; Yu, M.; Zhang, R.; Sun, Z.; Wang, Q. Controlled Synthesis of Ag_2Te@Ag_2S Core-Shell Quantum Dots with Enhanced and Tunable Fluorescence in the Second Near-Infrared Window. *Small* **2020**, *16*, 2001003.
41. Chen, P.; Qu, F.; Chen, S.; Li, J.; Shen, Q.; Sun, P.; Fan, Q. Bandgap Modulation and Lipid Intercalation Generates Ultrabright D-A-D-Based Zwitterionic Small-Molecule Nanoagent for Precise NIR-II Excitation Phototheranostic Applications. *Adv. Funct. Mater.* **2022**, *32*, 2208463.
42. Liu, S.; Xu, W.; Li, X.; Pang, D. W.; Xiong, H. BOIMPY-based NIR-II fluorophore with high brightness and long absorption beyond 1000 nm for in vivo bioimaging: synergistic steric regulation strategy. *ACS Nano* **2022**, *16*, 17424-17434.
43. Li, Y.; Cai, Z.; Liu, S.; Zhang, H.; Wong, S. T. H.; Lam, J. W. Y.; Kwok, R. T. K.; Qian, J.; Tang, B. Z. Design of AIEgens for near-infrared IIb imaging through structural modulation at molecular and morphological levels. *Nat. Commun.* **2020**, *11*, 1255.
44. Li, K.; Duan, X.; Jiang, Z.; Ding, D.; Chen, Y.; Zhang, G.-Q.; Liu, Z. J-Aggregates of Meso-[2.2]Paracyclophanyl-Bodipy Dye for NIR-II Imaging. *Nat. Commun.* **2021**, *12*, 2376.
45. Li, Y.; Zha, M.; Kang, T.; Li, C.; Wu, X.; Wang, S.; Lu, S. B.; Lee, Y. S.; Wu, Y. R.; Ni, J. S.; Li, K. Promoted NIR-II Fluorescence by Heteroatom-Inserted Rigid-Planar Cores for Monitoring Cell Therapy of Acute Lung Injury. *Small* **2022**, *18*, e2105362.
46. Ji, A.; Lou, H.; Qu, C.; Lu, W.; Hao, Y.; Li, J.; Wu, Y.; Chang, T.; Chen, H.; Cheng, Z. Acceptor Engineering for NIR-II Dyes with High Photochemical and Biomedical Performance. *Nat. Commun.* **2022**, *13*, 3815.
47. Zhang, X.; Li, L.; Ren, Y.; Li, M.; Tang, Y., Organic NIR-II Nanofluorophore with Ultrahigh Quantum Yield for Vessels Imaging and Fluorescence Image-Guided Surgery. *Adv. Funct. Mater.* **2025**, *35*, 2413341.
48. Akbarian, M.; Chen, I. N.; Lu, P. H.; Do, Q. T.; Tzeng, S. F.; Chou, H. H.; Chen, S. H. Chaperone/Polymer Complexation of Protein-Based Fluorescent Nanoclusters against Silica Encapsulation-Induced Physicochemical Stresses. *Biomacromolecules* **2024**, *25*, 6515-6525.
49. Hembury, M.; Beztsinna, N.; Asadi, H.; van den Dikkenberg, J. B.; Meeldijk, J. D.; Hennink, W. E.; Vermonden, T. Luminescent Gold Nanocluster-Decorated Polymeric Hybrid Particles with Assembly-Induced Emission. *Biomacromolecules* **2018**, *19*, 2841-2848.
